# Informing Adults With Back Pain About Placebo Effects: Randomized Controlled Evaluation of a New Website With Potential to Improve Informed Consent in Clinical Research

**DOI:** 10.2196/jmir.9955

**Published:** 2019-01-17

**Authors:** Felicity L Bishop, Maddy Greville-Harris, Jennifer Bostock, Amy Din, Cynthia A Graham, George Lewith, Christina Liossi, Tim O'Riordan, Peter White, Lucy Yardley

**Affiliations:** 1 Department of Psychology University of Southampton Southampton United Kingdom; 2 Institute of Psychiatry, Psychology & Neuroscience King's College London London United Kingdom; 3 Centre for Innovation & Leadership in Health Sciences School of Health Sciences University of Southampton Southampton United Kingdom; 4 Primary Care and Population Sciences Faculty of Medicine University of Southampton Southampton United Kingdom; 5 Zemedia Southampton United Kingdom; 6 School of Psychological Science Faculty of Life Sciences University of Bristol Bristol United Kingdom

**Keywords:** placebos, placebo effects, informed consent, research ethics, health knowledge, attitudes, practice, internet

## Abstract

**Background:**

Placebo effects and their underpinning mechanisms are increasingly well understood. However, this is poorly communicated to participants in placebo-controlled trials. For valid informed consent, participants should be informed about the potential benefits and risks of participating in placebo-controlled trials. Existing information leaflets often fail to describe the potential benefits and adverse effects associated with placebo allocation. This study tested the effects of a new website designed to inform patients about placebo effects (The Power of Placebos, PoP). PoP was designed using qualitative methods in combination with theory- and evidence-based approaches to ensure it was engaging, informative, and addressed patients’ concerns.

**Objective:**

This study aimed to test the effects of PoP, compared with a control website, on people’s knowledge about placebo and the ability to make an informed choice about taking part in a placebo-controlled trial.

**Methods:**

A total of 350 adults with back pain recruited from 26 general practices in Southern England participated in this Web-based study. Participants were randomly assigned to PoP (which presented scientifically accurate information about placebo effects in an engaging way) or a control website (based on existing information leaflets from UK trials). Participants self-completed Web-based pre- and postintervention questionnaire measures of knowledge about placebo effects and preintervention questionnaire measures of attitudes toward and intentions to participate in a placebo-controlled trial. The 2 primary outcomes were (1) knowledge and (2) informed choice to take part in a placebo-controlled trial (computed from knowledge, attitudes, and intentions).

**Results:**

After viewing PoP, participants had significantly greater knowledge about placebos (mean 8.28 [SD 1.76]; n=158) than participants who viewed the control (mean 5.60 [SD 2.24]; n=174; *F*_1,329_=173.821; *P*<.001; *η*^2^=.346). Participants who viewed PoP were 3.16 times more likely than those who viewed the control to make an informed choice about placebos (χ^2^_1_=36.5; *P*<.001).

**Conclusions:**

In a sample of adults with back pain, PoP increased knowledge and rates of informed choice about placebos compared with a control website. PoP could be used to improve knowledge about placebo effects in back pain. After essential further development and testing in clinical trial settings, it could support informed consent in placebo-controlled trials.

## Introduction

### Background

Placebo-controlled trials remain the gold standard for establishing the efficacy of new pharmacological and other interventions and are often used in pain medicine. According to the Declaration of Helsinki, investigators should obtain a priori voluntary informed consent from participants after informing them about “the anticipated benefits and potential risks of the study and the discomfort it may entail” (item 26, [1]). Although participant information leaflets typically focus on the benefits and risks of the new intervention being trialed [2], placebos themselves can trigger both beneficial and adverse effects in a range of conditions, particularly pain [3-7]. Patients in a trial who receive the placebo may thus experience placebo and/or nocebo effects but are unlikely to have been informed about them in advance. Arguably, it should be standard practice to inform patients in placebo-controlled trials about the placebo and its possible benefits and adverse effects [8].

Current information provision about placebo interventions in clinical trials is likely to be inadequate and thus jeopardizes the validity of informed consent. Qualitative studies and surveys suggest that members of the public and trial participants often have misunderstandings and partial knowledge about placebos and their effects [9,10]. For example, trial participants often believe that placebo effects are fake or illusory and that people who respond to placebos are gullible or foolish [10-12]. Such beliefs are inconsistent with the scientific literature on placebo effects and may: deter people from volunteering for trials [13]; contribute to patient anxiety about placebo effects [11]; and/or make it difficult for participants to make sense of a personal placebo response when debriefed to placebo allocation at the end of a trial [14,15]. Furthermore, only a small minority of people understand that placebos can have adverse effects; this has been found consistently across surveys of trial participants [9] and patients [16-18]. These beliefs are also unlikely to be challenged by existing patient information leaflets, which barely mention the placebo; a content analysis of participant information leaflets from major UK-based placebo-controlled trials found that only 1 of the 45 leaflets studied explicitly stated that patients might experience beneficial effects from the placebo, only 4 leaflets explicitly stated that patients might experience adverse effects from the placebo, and 8 leaflets explicitly stated that the placebo treatment was either undesirable or ineffective [2]. This paucity of information about placebos is probably widespread, as studies of patient information leaflets have reported similar findings, for example, in Finland [19], Spain [20], and internationally [21].

Preliminary evidence suggests it is possible to improve people’s understandings of placebos [22,23], but effects in well-defined clinical populations and on key outcomes such as informed choice have not been evaluated. Web-based resources could usefully augment or improve existing paper-based information leaflet because websites (1) are increasingly popular with health consumers [24,25]; (2) easily incorporate interactive features [26], which can enhance engagement and effective education [27]; (3) are easily and cheaply disseminated for widespread access [26]; and (4) can be readily adapted and/or tailored for use in different clinical trials [26].

### Objectives

A Web-based experiment was conducted to compare a newly developed, scientifically accurate, and engaging website about placebos—The Power of Placebos (PoP) [28]—with a control website based on existing patient information about placebos. PoP was designed for and tested in adults with back pain because placebo analgesia is well documented in this population [3,5,29,30]. It was important to focus on 1 condition as the nature of and mechanisms underpinning placebo effects differ by disease [31]. Back pain is a major public health concern [32] as it is the leading cause of disability in most countries [33]. The most effective therapies appear to involve exercise and education [34], although 1 study has found improvements in back pain after open-label placebo treatment [35]. The hypotheses were that PoP would increase knowledge about placebos, enable more patients to make an informed choice about having placebos, and encourage people to believe that placebos can have credible analgesic and adverse effects.

## Methods

### Design

This Web-based study tested the effects of a newly developed interactive website (PoP) on patients’ knowledge, informed choice, and beliefs about placebos. Participants were randomized automatically by the overarching study website to view either PoP or the control website. They also saw a separate website about acupuncture (again either a newly developed one or a control), as this study was part of a larger 2 × 2 factorial trial (for details, see Multimedia Appendix 1). As there were no effects of the acupuncture website manipulation on patients’ knowledge, informed choice, and beliefs about placebos, this study reports the PoP versus control comparison for the placebo website only. The equivalent comparison for the acupuncture website is being reported separately.

Furthermore, 2 volunteers, with personal experience of back pain and clinical research, acted as patient advisors in this study. Research has shown that such “patient public involvement” in research can enhance the design and conduct of studies [36]. Both our volunteers participated in team meetings, which involved discussions and decision making around study design, procedures, and conduct. The patient advisors also helped to finalize recruitment materials and contributed to website design, for example, by providing feedback on prototypes of PoP. Of the 2 advisors, 1 advisor also chose to contribute to the process of writing up for publication.

Ethical approval was sought and obtained from the University of Southampton (Ethics ID: 12323) and the NHS NRES (National Health Service National Research Ethics Service) Committee East of England-Hatfield (Research Ethics Committee reference: 14/EE/1176).

### Participants and Recruitment

**Figure 1:**
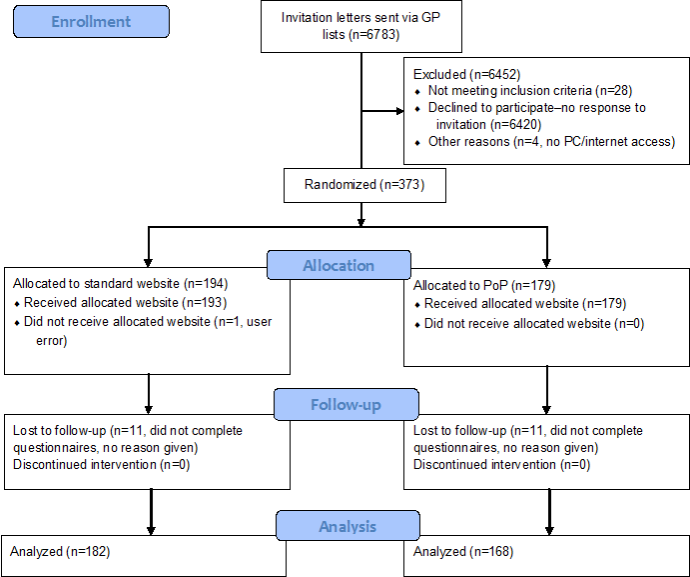
Flow of participants through the study. GP: general practitioner; PoP: The Power of Placebos.

Adults aged 18 years and older with a recent history of back pain (within 3 years) as documented by their general practitioner (GP) were recruited via 26 general practices in South West England between December 2014 and March 2015. General practice staff conducted database searches and mailed study invitation packs to eligible patients. Invitation packs contained a cover letter and information sheet including the study website address. Participants needed to be computer literate to self-enroll in the study and complete it independently (technical support was available from the researchers by telephone or email if needed). People with needle phobia (important for the acupuncture websites) or unable to complete questionnaires in English were excluded. Figure 1 shows the flow of participants through the study.

An a priori power calculation was conducted using G*Power (Heinrich Heine Universitat Dusseldorf) [37]. Assuming an effect size *f*=0.15 (based on unpublished pilot data), power 0.8, and alpha .05 for a factorial analysis of variance (ANOVA) required a total of 351 participants, and assuming 5% dropout required 369 patients to be randomized.

### Interventions

Overall, 2 websites about placebo effects were developed for this study: a person-based website and a control website. Both websites were explicitly labeled as developed at the University of Southampton and included identical brief biographies of the research team. Participants were not told which website they were viewing. Both websites presented information about placebo effects and were created using LifeGuide software, a package developed for researchers to design, build, and trial Web-based interventions [38]. Guidance for developing patient-focused health information was followed to ensure both websites were understandable, readable, and accessible [39]. The person-based website was longer than the control website (10 pages vs 5 pages). Table 1 compares the content and formats of each website. No changes to the websites were made during this study.

**Table 1:** Summary comparison of The Power of Placebos (PoP) and control websites.

Content and format of websites		PoP	Control
**Contents**			
	Defining placebos	✓^a^	✓
	Potential benefits of placebo	✓	✓
	Potential adverse effects of placebo	✓	X^b^
	Patients’ experiences of placebos	✓	X
	Common concerns about placebos	✓	X
	Debunking myths that placebo responders are malingerers or gullible	✓	X
	Mechanisms underpinning placebo effects	✓	X
	Placebos in placebo-controlled trials	✓	✓
	Placebos in clinical practice	✓	X
**Formats**			
	Text	✓	✓
	Images	✓	✓
	Film	✓	X
	Audio clips	✓	X

^a^Tick indicates feature is present in the website.

^b^Cross indicates feature is absent in the website.

#### The Power of Placebos Website

**Figure 2:**
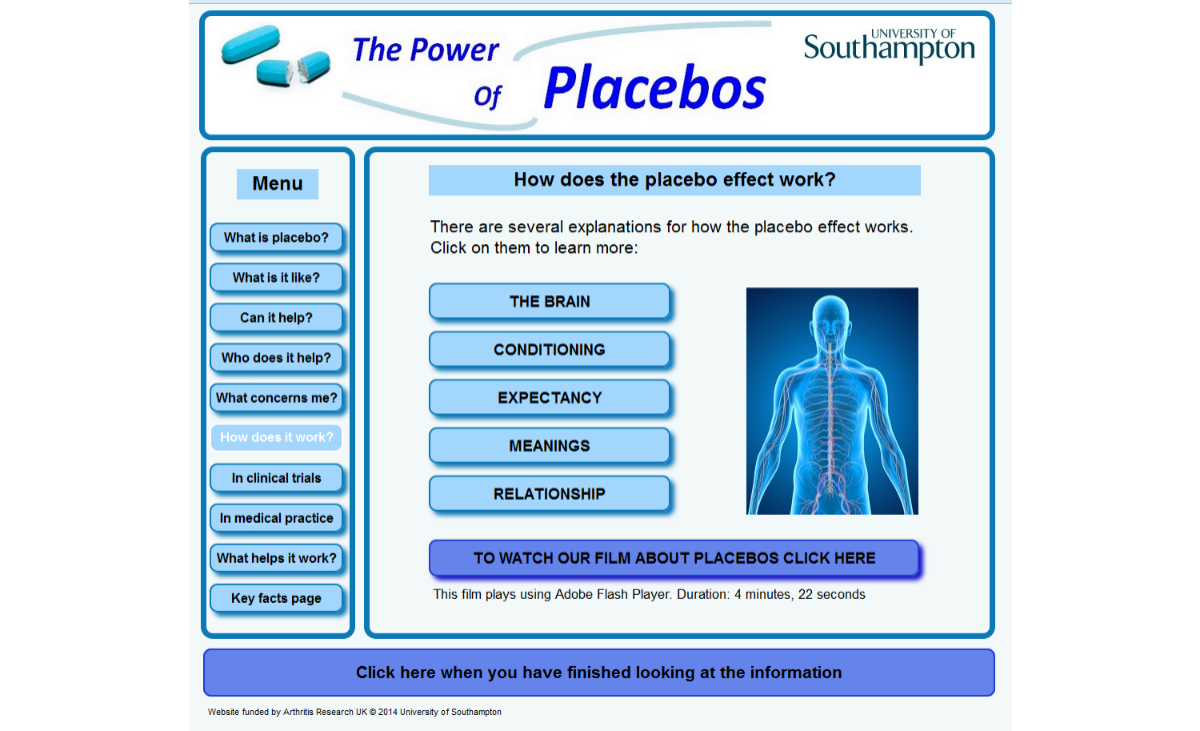
Screenshot from the Power of Placebos website: How do placebos work?

PoP was developed using a rigorous approach derived from person-based intervention development, which incorporates evidence and theory [40-42]. This entailed extensive intervention planning, drawing on existing evidence and theory about placebo effects and how to change patients’ health beliefs and behaviors, and conducting qualitative think-aloud interviews to develop and refine the website. The content was based on published scientific evidence [3,4,31,43] and targeted theoretically informed constructs (primarily knowledge but also attitudes, subjective norms, and perceived behavioral control, derived from the Theory of Planned Behavior [44]). For a full description of the development process and the resulting website, refer to the study by Greville-Harris et al [28]. Figure 2 shows an example page, and screenshots showing additional pages are available in Multimedia Appendix 2. A completed TIDieR (template for intervention description and replication) checklist in Multimedia Appendix 3 presents full information about the description of PoP.

#### The Control

**Figure 3:**
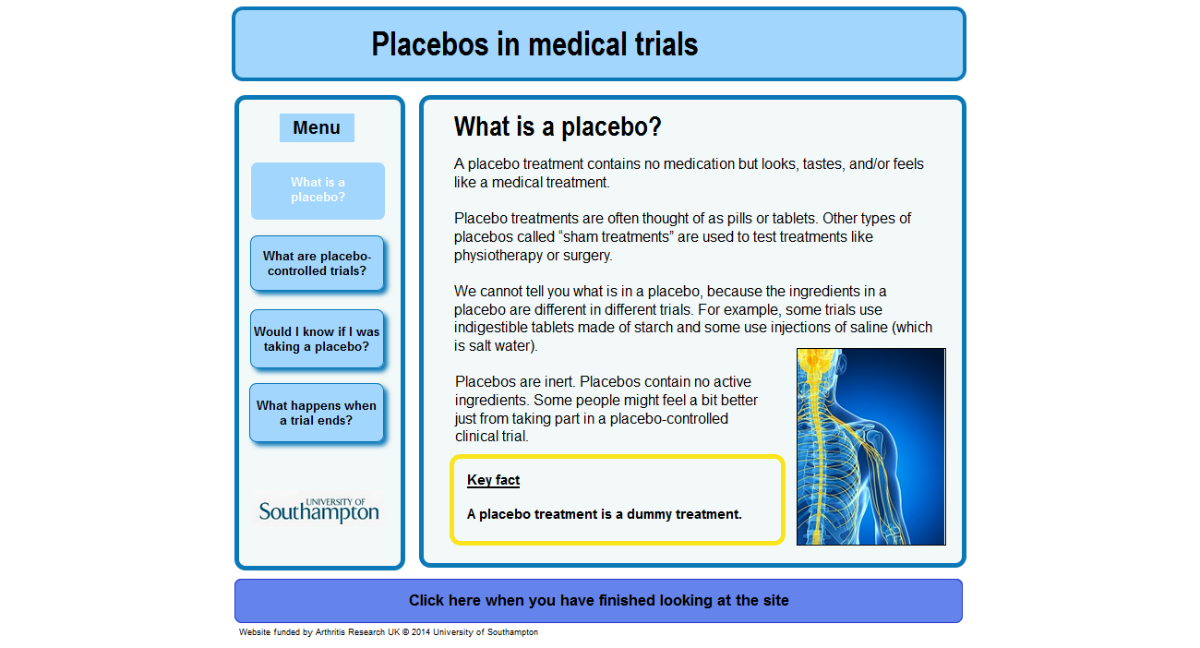
Screenshot from control webpage.

The control website was based on the limited information about placebos that is included in some UK patient information leaflets for placebo-controlled clinical trials. Consistent with common practice among those leaflets that provide any information about placebos [2], it provided only minimal detail about the possible effects of placebos and did not explain their mechanisms of action. Figure 3 shows an example page. Screenshots of the entire control website are available in Multimedia Appendix 4.

### Measures

#### Participants’ Characteristics

Items selected from the recommended minimum dataset for back pain [45] were used to assess clinical characteristics (pain duration, pain frequency, pain intensity, pain catastrophizing, and pain spread to legs), pain-related legal claims, disability benefits or compensation (all single items), and pain functioning and interference (4-item scales with excellent internal consistency in this sample; Cronbach alphas .96 and .92, respectively). Single items assessed ethnicity, age, gender, and education level.

#### Primary Outcomes

Primary outcomes were knowledge and informed choice about placebos. Informed choice was selected as an analogy to informed consent because the website was being tested outside the context of a placebo-controlled trial. Informed choice is a composite measure based on knowledge, attitudes, and intentions to try placebo, the components of which were measured after participants had finished viewing the website.

Placebo knowledge was assessed using a 10-item knowledge quiz, comprising true-false questions developed and validated in preparation for this study. These 10 items were selected from a larger pool of 15 items pilot tested in a Web-based study with a community-based sample of 210 adults with a history of back pain [18]. The 10 items most commonly answered incorrectly by the community-based sample were chosen for this study (eg, “A placebo pill can have side effects” and “Placebo pills can help to treat pain conditions”—both are true). The knowledge score is the total number of items answered correctly. The quiz was completed before and after viewing the websites. The pre- and postadministrations had acceptable internal consistency in this sample (Cronbach alphas .66 and .79, respectively).

Making an informed choice can be understood as choosing to act in a way that is based on one’s knowledge and one’s values [46-48]. To make an informed choice, a person needs to have an accurate understanding of the options available, have formed an opinion about the options based on their personal values, and make a decision (or otherwise act in a way) that is consistent with their knowledge and values [46-48]. Although it can be argued that knowledge alone is sufficient for informed choice, attitudes are incorporated in our chosen definition as an attempt to reflect the role of personal values in making health decisions that are optimized for the individual concerned. If decisions were based on knowledge alone, people could still make decisions that are inconsistent with their personal values and so could be considered sub-optimal for them as individuals. By choosing a definition of informed choice that incorporates attitudes, we are better able to model the role of personal values in decision making. From this perspective, an informed choice to have a placebo requires knowledge about the possible beneficial and adverse effects of placebos, a positive attitude to placebos, and a decision to try a placebo. An informed choice not to have a placebo requires knowledge about the possible beneficial and adverse effects of placebos, a negative attitude to placebos, and a decision not to try a placebo.

In this study, knowledge was measured using the placebo knowledge quiz. Attitudes were measured using 4 items derived following the Theory of Planned Behavior guidelines for measuring attitudes [49], for example, “having a placebo treatment would be good.” Behavioral intentions were used as a proxy for behavior and were measured using 3 items derived following the same guidelines [49], for example, “if given the opportunity, I intend to have a placebo treatment.” Items assessing attitudes and intentions were measured on 7-point Likert-type scales labeled strongly disagree to strongly agree, and scores across constituent items were averaged. The scales had good internal consistency (Cronbach alphas .97 and .87, respectively).

Participants were categorized as making an informed choice or not based on their attitude (split by scale midpoint to produce 2 groups with positive and negative attitudes), intention to have treatment (split by scale midpoint to produce 2 groups with positive and negative intentions), and their knowledge score (based on median split to produce 2 groups with high and low knowledge). Participants were categorized as making an informed choice if they scored above the median on knowledge and either (a) above the scale midpoint on both attitudes and intentions or (b) below the scale midpoint on both attitudes and intentions. All other participants were categorized as not making an informed choice. Although there are drawbacks of computing composite measures in this way (see Discussion), this approach enabled us to follow published definitions of informed choice to create a primary outcome measure with good face validity and is consistent with previous studies of informed choice in other settings [46-48].

#### Secondary Outcomes

Secondary outcomes were participants’ beliefs about placebos and willingness to try a placebo. These were completed after participants had finished viewing the website.

Overall, 4 dimensions of beliefs about placebos were measured using the previously validated 4-item subscales of the Low Back Pain Treatment Beliefs Questionnaire [50]: concerns (eg, “I have concerns about having placebos for my back pain”), individual fit (eg, “I am confident placebos would be a suitable treatment for my back pain”), expectancy (eg, “Placebos can work well for people with back pain”), and credibility (eg, “Generally, placebos are a believable therapy for back pain”). The Low Back Pain Treatment Beliefs Questionnaire was validated in a Web-based study [50]. All items used 5-point Likert-type response scales labeled strongly disagree to strongly agree. All subscales had good internal consistency in this sample (Cronbach alpha for concerns=.72, individual fit=.91, expectancy=.94, and credibility=.84).

A single yes or no item asked whether participants would be “willing to have placebo treatment.” As participants could take breaks from viewing the website and return later, an item was also included to assess whether participants had “looked up additional information about placebo during breaks from the study.”

### Procedure

On accessing the study website at a time and location of their choice (eg, at home), not in the presence of a researcher, participants viewed the information sheet and indicated consent to take part by clicking a button (see Multimedia Appendix 5 for information sheet). Participants were then asked 3 mandatory screening questions assessing age, current or recent back pain, and needle phobia. Those not meeting the associated inclusion criteria were directed to an exit page. Those passing the screening questions entered their email address and created a password for the website, which allowed participants to take breaks when desired. Participants then completed the 10-item placebo knowledge quiz to assess baseline knowledge before being presented with the series of 2 websites (placebo and acupuncture) appropriate to their randomization group. Participants could take breaks, log out and return to the study later, and stop viewing each website whenever they wanted (“click here when you have finished looking at the information” was available on every page). Directly after viewing the websites, participants completed the primary and secondary outcome measures and measures of sociodemographic and clinical characteristics. All measures were completed on the study website. Finally, participants were directed to a debriefing page with further information about the study and links to other resources. Everyone who completed the study was emailed a £10 online shopping voucher.

### Statistical Analysis

Data were downloaded from LifeGuide and imported into IBM SPSS Statistics v22 for analysis. The proportion of missing data was small (<5% on any 1 variable) but was not missing completely at random (MCAR; Little MCAR test: χ^2^_7845_=8279.7; *P*<.001), suggesting imputation might be inappropriate but unlikely to alter the results [51]. All analyses were repeated excluding missing data and then imputing missing values with the expectation-maximization algorithm. The results were the same, and the reported analyses used all available data with no imputation.

Pearson’s chi-square test compared the proportions of people (a) making an informed choice and (b) willing to have a placebo, between PoP and the control website. ANOVAs tested the effects of PoP compared with control on postintervention measures of knowledge and treatment perceptions. Analyses of covariance were then computed adjusting for baseline knowledge [52], which was the only baseline variable to differ between the groups.

## Results

### Participants’ Characteristics

The final sample comprised 350 adults, of whom the majority were female, white British, and educated up to at least 18 years (see Table 2). The average age of participants was 47.88 years (SD 15.8; control group mean age 48.80 years [SD 15.9]; PoP group mean age 46.88 years [SD 15.6]). Almost all participants (308/350, 88.0%) reported having back pain in the last week, and on average, they reported pain interfering with daily activities between *a little bit* and *somewhat* and being able to perform functional tasks such as chores or walking *with a little difficulty*. Participants’ back pain was typically longstanding (157/350, 45.1% had onset over 5 years ago), affected them daily or almost daily (133/350, 38.0%), and was of moderate intensity (mean 4.75 on a 10-point scale; control group mean 4.79 [SD 2.46]; PoP group mean 4.71 [SD 2.40]). Pain interference in the past week was also moderate (control group mean 2.58 [SD 1.29]; PoP group mean 2.58 [SD 1.25]; on a 5-point scale) as was current pain functioning (control group mean 1.98 [SD 0.96]; PoP group mean 2.06 [SD 1.02]; on a 5-point scale). Approximately, one-third (126/350, 36.0%) reported pain catastrophizing. Very few participants (2 per group) reported looking up additional information about placebo effects during the study. The groups did not differ significantly on any of these measures (all *P* values >.05), except baseline knowledge about placebo, on which the PoP group scored slightly higher (mean 6.21 [SD 2.14]) than the control group (mean 5.72 [SD 2.20]; *t*_340_=−2.08; *P*=.04).

### Knowledge, Attitudes, and Intentions

There was a significant main effect of website on placebo knowledge: after viewing the website, people who viewed PoP had higher knowledge about placebo effects than people who viewed the control website (Table 3). On average, people who viewed PoP also had less positive attitudes and more positive intentions toward placebo effects than people who viewed the control website, but these effects were much smaller than the effect on knowledge (Table 3).

### Informed Choice

Table 4 shows how participants were classified as making an informed choice or not according to their knowledge, attitudes, and intentions. There was a significant association between website and informed choice about placebo (*χ*^*2*^_1_=36.5; *P*<.001). Almost half of people (66/146, 45.2%) who viewed PoP made an informed choice about placebos compared with 14.3% (24/168) of people who viewed the control website. Thus, people who viewed PoP were 3.16 times more likely than those who viewed the control website to make an informed choice about placebos. The most common pattern of scores on components of informed choice was to have negative intentions of having placebos, *and* negative attitudes toward placebos, *and* low knowledge about placebos, a pattern displayed by 27.4% of all participants, 41.1% of participants who viewed the control website, and 11.6% of participants who viewed PoP.

### Treatment Beliefs

After viewing the website, people who viewed PoP had significantly more positive beliefs that placebo treatment was a good fit for them, had significantly higher expectations of benefit from placebo, and perceived placebo treatment as significantly more credible than people who viewed the control website (Table 5). Level of concerns about placebo treatment did not differ between the groups.

### Willingness to Try Placebo

There was a significant effect of website on willingness to try a placebo (χ^2^_1_=10.1; *P*=.001). More than half of participants (59.3%, 99/167) who had viewed PoP compared to 42.2% (76/180) of those who had viewed the control website said they would be willing to try a placebo. Thus, people who viewed PoP were 1.41 times more likely than those who viewed the control website to be willing to try a placebo.

**Table 2:** Participants’ characteristics by group.

Characteristic and category			Frequency, n (%)		
			Whole sample (N=350)	Control website (n=182)	PoP^a^ (n=168)
**Sociodemographic characteristics**					
	**Gender**				
		Female	197 (56.3)	102 (56.0)	95 (56.5)
	**Ethnicity**				
		White British	311 (88.9)	166 (91.2)	145 (86.3)
		White (any other)	16 (4.6)	7 (3.8)	9 (5.4)
		Asian or Asian British	4 (1.2)	1 (0.5)	3 (1.8)
		Mixed ethnicity	2 (0.6)	0 (0.0)	2 (1.2)
		Black or black British	2 (0.6)	1 (0.5)	0 (0.0)
	**Education**				
		Did not complete secondary school	19 (5.4)	13 (7.1)	6 (3.6)
		Secondary school	89 (25.4)	48 (26.4)	41 (24.4)
		Sixth form or college (aged 16-18 years)	106 (30.3)	61 (33.5)	45 (26.8)
		Undergraduate study	98 (28.0)	43 (23.6)	55 (32.7)
		Postgraduate study	35 (10.0)	16 (8.8)	19 (11.3)
**Clinical characteristics**					
	**Time since pain onset**				
		Up to 1 year	71 (20.4)	29 (15.9)	42 (25.0)
		1 to 5 years	105 (30.2)	57 (31.3)	48 (28.6)
		>5 years	157 (45.1)	83 (45.6)	74 (44.0)
	**Pain frequency in past 6 months**				
		Every day or nearly every day	133 (38.0)	70 (38.5)	63 (37.5)
		More than half the days	85 (24.3)	40 (22.0)	45 (26.8)
		Less than half the days	102 (29.1)	53 (29.1)	49 (29.2)
	Disability or compensation benefits		16 (4.6)	7 (3.8)	9 (5.4)
	Legal claim related to back		4 (1.1)	1 (0.5)	3 (1.8)
	Pain spread to leg or legs in past 2 weeks		142 (40.6)	73 (40.1)	69 (41.1)
	Pain catastrophizing		126 (36.0)	66 (36.3)	60 (35.7)
	Previous participation in a placebo-controlled trial		6 (1.7)	4 (2.2)	2 (1.2)
	Looked up additional information about placebos during the study		4 (1.1)	2 (1.1)	2 (1.2)

^a^PoP: The Power of Placebos.

**Table 3:** Postintervention knowledge, attitudes, and intentions toward placebos by group.

Measure	Control website		The Power of Placebos		Comparison across websites^a^		*η_p_* ^2^
	Mean (SD)	n (%)	Mean (SD)	n (%)	*F* test (*df*)	*P* value	
Knowledge^b^	5.60 (2.24)	174 (52)	8.28 (1.76)	158 (48)	173.821 (1,329)	<.001	0.346
Attitudes^c^	3.89 (1.28)	173 (53)	3.24 (1.31)	156 (47)	15.779 (1,326)	<.001	0.046
Intentions^c^	2.58 (1.65)	172 (52)	3.34 (1.87)	161 (48)	13.264 (1,330)	<.001	0.039

^a^Models adjusted for baseline knowledge.

^b^Possible score range 0 to 10 (10=high knowledge).

^c^Possible score range 1 to 7 (7=positive attitudes or intentions).

**Table 4:** Informed choice categories.

Informed choice	Knowledge	Attitude	Intentions	Whole sample (N=314), n (%)	Control website (n=168), n (%)	PoP^a^ (n=146), n (%)
No	Low	Positive	Negative	28 (8.9)	25 (14.9)	3 (2.1)
No	Low	Negative	Positive	17 (5.4)	14 (8.3)	3 (2.1)
No	Low	Positive	Positive	29 (9.2)	20 (11.9)	9 (6.2)
No	Low	Negative	Negative	86 (27.4)	69 (41.1)	17 (11.6)
No	High	Positive	Negative	50 (15.9)	11 (6.5)	39 (26.7)
No	High	Negative	Positive	14 (4.5)	5 (3.0)	9 (6.2)
Yes	High	Positive	Positive	52 (16.6)	9 (5.4)	43 (29.5)
Yes	High	Negative	Negative	38 (12.1)	15 (8.9)	23 (15.8)

^a^PoP: The Power of Placebos.

**Table 5:** Postintervention treatment beliefs by group.

Treatment belief	Control website		PoP^a^		Comparison across websites^b^		*η_p_* ^2^
	Mean (SD)	n (%)	Mean (SD)	n (%)	*F* test (*df*)	*P* value	
Concerns^c^	2.40 (0.82)	173 (51)	2.29 (0.84)	163 (49)	0.810 (1,333)	0.37	0.002
Individual fit^c^	2.19 (0.91)	172 (52)	2.76 (1.03)	159 (48)	23.728 (1,328)	<.001	0.067
Expectancy^c^	2.59 (1.00)	172 (52)	3.40 (0.88)	161 (48)	58.657 (1,330)	<.001	0.151
Credibility^c^	2.43 (0.90)	173 (52)	3.06 (0.91)	162 (48)	36.529 (1,332)	<.001	0.099

^a^PoP: The Power of Placebos.

^b^Models adjusted for baseline knowledge.

^c^Possible score range 1 to 5 (5=positive beliefs or fewer concerns about placebo).

## Discussion

### Principal Findings

This study tested the effects of a new person-based website about placebo effects, PoP, on adults with recent back pain, by comparing it with a control website based on existing written UK patient information leaflets. Participants who viewed PoP had greater increases in knowledge about placebos and were 3 times more likely to make an informed choice about placebos compared with participants who viewed the control website. On average, participants who viewed PoP answered 2 more knowledge quiz items correctly (out of a total of 10 items). Compared with the control website, PoP also led participants to perceive placebos as more credible, more effective, and more suitable for them personally and to be more willing to try a placebo in the future.

Other studies have also successfully modified people’s beliefs and/or knowledge about placebos. For example, 1 study [22] compared a brief educational intervention comprising 5 slides presenting information about placebo effects and their mechanisms with a control intervention presenting information about the epidemiology, costs, and risk factors for musculoskeletal pain. Participants with chronic musculoskeletal pain were randomly assigned to 1 of the 2 interventions. Those who viewed the placebo educational intervention subsequently reported feeling more knowledgeable about placebo analgesia, seeing placebos as more active, more effective, and more acceptable in a range of hypothetical clinical scenarios [22]. Another study [23] compared 2 patient information leaflets about placebo-controlled randomized trials, one based on standard information and the other supplemented with additional information about placebos, their effects, and mechanisms of action. In an online randomized experiment, people with chronic illness who viewed the supplemented leaflet subsequently reported significantly higher expectations and perceptions of the credibility of placebo treatment for pain compared with those who viewed the standard information leaflet [23]. PoP is more comprehensive than educational and/or informational resources on placebo effects reported previously, and it was developed using a systematic approach described in detail elsewhere [28]. Similar to the studies by Kisaalita et al [22] and Bishop et al [23], this study has shown that information about placebos and placebo effects can lead patients to have more positive beliefs about placebos. This study has also shown effects on beliefs about placebos in a large sample of patients with a particular pain condition. Uniquely, PoP had a significant effect on objectively measured knowledge and informed choice, a close analogy to informed consent in both clinical and research settings. PoP also increased willingness to try a placebo, suggesting it might help facilitate recruitment to placebo-controlled randomized controlled trials (RCTs). Future work should test PoP in the context of a placebo-controlled trial, to ascertain whether it can indeed encourage more people to volunteer.

PoP could be readily adapted for use in other conditions, which, like pain, have well-documented placebo effects (such as irritable bowel syndrome [53] or depression [54]), and/or specific placebo-controlled RCTs. PoP could be adapted to improve patients’ understanding of open-label placebo interventions [35,55] and/or to educate patients about placebo effects and mind-body interactions more broadly. It would be interesting to examine its impact on willingness to use such interventions. It would also be interesting to explore its use in placebo-controlled surgery trials where there are large placebo effects [56]. However, it would not be appropriate to use PoP as part of participant information for trials in conditions in which placebo effects are poorly understood or rarely seen, making it important to consult up-to-date reviews of placebo effects before finalizing placebo information for any specific trial. Furthermore, before it is used in an RCT, it is important to test for any additional effects of PoP on trial variables, specifically the size of the placebo effect in both placebo and verum arms, the success of blinding, and patients’ reactions to debriefing. In this study, the person-based website led to increased expectations of effectiveness of a placebo. Positive outcome expectations are a key mechanism underpinning placebo effects in pain and other conditions [31]. Therefore, if PoP were to increase patients’ expectations of effectiveness in a placebo-controlled RCT, this could also lead to larger placebo effects and thus have implications for power calculations and the efficacy of the target treatment.

The use of PoP in RCTs could also have implications for successful blinding of patients to treatment allocation. Qualitative studies suggest that patients typically believe if they experience no side effects in a trial, then this means they are receiving the placebo, whereas if they do experience side effects, then this means they are receiving the verum intervention [12,57]. This logic is appealing but incorrect as patients in placebo groups regularly report benefits and adverse effects, particularly in pain trials [5,58]. As PoP increases patients’ knowledge about the positive and adverse effects of placebos, this could reduce patients’ confidence in a link between effects and treatment allocation, thus helping to maintain uncertainty and, therefore, blinding. Believing that placebos can have no effects might also lead patients to be surprised and/or distressed on being debriefed at the end of a trial and finding out they were in the placebo group [10,14,15,57]. Future research could use PoP to test whether providing more comprehensive information about placebos at the start of a trial can improve patients’ and investigators’ experiences of debriefing at the end of a trial.

### Strengths and Limitations

Strengths of this study include the systematic approach used to develop the person-based website, the choice of a Web-based modality and the use of a control intervention. PoP had previously been developed using person-based, theory-based, and evidence-based intervention designs [28,42]. This ensured it was engaging, persuasive, and based on current scientific evidence about the size and mechanisms of action of placebo effects. Compared with traditional paper-based patient information leaflets, creating a website enabled us to provide more detailed information in an accessible and engaging manner using a range of formats including text, audio, and film. Other studies of person-based digital interventions typically compare them with usual care (ie, nondigital) [59,60]. This study contributes a demonstration of the value of the person-based approach to intervention development as compared with a website based on standard written patient information.

Limitations stem from the choice of control, the sample, and the measure of informed choice. The use of a control website based on existing printed materials enabled a pragmatic evaluation of the potential impact of PoP but did not allow an evaluation of which components of the website were most important; for example, both websites included (different) text and images, but PoP also included audio clips and short films and was longer than the control website. It is, therefore, possible that the effects of PoP were because of these differences in sheer volume of information.

Online health information is accessible to a large majority—but not all—of the population: in 2015, 86% of UK households had internet access and 78% of adults accessed the internet daily or almost daily [61]. The generalizability of the study is somewhat limited as there was a very low uptake from the initial mail out via GP surgeries (leading to possible selection bias) and only people with self-reported recent back pain were included. It is not possible to compare the participants with all patients who were invited to take part as no information could be ascertained about the latter group. As we invited patients who had consulted their GP with back pain as much as 3 years ago, it is possible that many of the nonresponders were no longer seeking treatment for back pain, and therefore, this study was not relevant to them. The inclusion criteria do not directly map onto current definitions of acute or chronic back pain but were chosen to capture a group of people for whom PoP might be of interest. The website should have similar effects on other groups of people with painful conditions, but this could be tested in future studies. The final sample size fell short of the a priori sample size calculation by 1 participant, but as almost all the results were statistically significant, this did not seem to have an impact on the findings.

The outcome measures were previously validated and included an assessment of objective knowledge and an assessment of informed choice, which is particularly relevant when considering the potential use of the website to inform volunteers for RCTs. However, the conceptual strength of measuring informed choice must be balanced by an acknowledgment of the statistical limitations of the loss of data associated with this particular outcome measure, as it was derived (following published guidelines) by dichotomizing 3 continuous variables. Finally, it must be noted that the participants in this study were asked about intentions and willingness to take part in a placebo-controlled trial without actually being invited to take part in a specific trial. This is a limitation for at least two reasons. First, intentions and willingness to do something do not always translate into actual behavior [62]. Second, placebo-controlled trial participants receive information about the trial treatment and procedures as well as about the placebo, and they have to decide whether they would be willing to receive either, not just one, of these interventions. Future studies should, therefore, examine the use of enhanced information resources such as the PoP website in the context of an actual placebo-controlled trial.

### Conclusions

In conclusion, the person-based website, PoP, increased knowledge, led more participants to make an informed choice, and enhanced positive beliefs about placebos in a sample of adults with recent back pain recruited from primary care. It could be used to increase levels of understanding about placebo effects among the general public. In future, PoP could be adapted and used to support informed consent and recruitment to placebo-controlled clinical trials, but first, its effects on recruitment and trial outcomes should be investigated in the context of a placebo-controlled trial. This study provides initial evidence suggesting that the person-based method of developing Web-based interventions—combining extensive qualitative research with evidence-based and theory-based methods [42]—could be used to improve informed consent materials in clinical research. Further work is needed to confirm its utility in the development of materials to support the ethical conduct of clinical research.

## Appendix

Multimedia Appendix 1 Description of the factorial trial design.

Multimedia Appendix 2 Screenshots showing additional pages from PoP.

Multimedia Appendix 3 A completed TIDieR checklist with full information about the description of PoP.

Multimedia Appendix 4 Screenshots of the entire control website.

Multimedia Appendix 5 Information sheet and consent form for study participants.

## Abbreviations

ANOVA: analysis of variance
GP: general practitioner
MCAR: missing completely at random
PoP: The Power of Placebos
RCT: randomized controlled trial
TIDieR: template for intervention description and replication

## References

[1] World Medical Association (2013). World Medical Association Declaration of Helsinki: ethical principles for medical research involving human subjects. J Am Med Assoc.

[2] Bishop FL, Adams AE, Kaptchuk TJ, Lewith GT (2012). Informed consent and placebo effects: a content analysis of information leaflets to identify what clinical trial participants are told about placebos. PLoS One.

[3] Finniss DG, Kaptchuk TJ, Miller F, Benedetti F (2010). Biological, clinical, and ethical advances of placebo effects. Lancet.

[4] Benedetti F, Amanzio M (2011). The placebo response: how words and rituals change the patient's brain. Patient Educ Couns.

[5] Hróbjartsson A, Gøtzsche PC (2010). Placebo interventions for all clinical conditions. Cochrane Database Syst Rev.

[6] Forsberg JT, Martinussen M, Flaten MA (2017). The placebo analgesic effect in healthy individuals and patients: a meta-analysis. Psychosom Med.

[7] Howick J, Friedemann C, Tsakok M, Watson R, Tsakok T, Thomas J, Perera R, Fleming S, Heneghan C (2013). Are treatments more effective than placebos? A systematic review and meta-analysis. PLoS One.

[8] Blease CR, Bishop FL, Kaptchuk TJ (2017). Informed consent and clinical trials: where is the placebo effect?. Br Med J.

[9] Criscione LG, Sugarman J, Sanders L, Pisetsky DS, St Clair EW (2003). Informed consent in a clinical trial of a novel treatment for rheumatoid arthritis. Arthritis Rheum.

[10] Bishop FL, Jacobson EE, Shaw JR, Kaptchuk TJ (2012). Scientific tools, fake treatments, or triggers for psychological healing: how clinical trial participants conceptualise placebos. Soc Sci Med.

[11] Kaptchuk TJ, Shaw J, Kerr CE, Conboy LA, Kelley JM, Csordas TJ, Lembo AJ, Jacobson EE (2009). “Maybe I made up the whole thing”: placebos and patients' experiences in a randomized controlled trial. Cult Med Psychiatry.

[12] White P, Bishop FL, Prescott P, Scott C, Little P, Lewith G (2012). Practice, practitioner, or placebo? A multifactorial, mixed-methods randomized controlled trial of acupuncture. Pain.

[13] Welton AJ, Vickers MR, Cooper JA, Meade TW, Marteau TM (1999). Is recruitment more difficult with a placebo arm in randomised controlled trials? A quasirandomised, interview based study. Br Med J.

[14] Bishop FL, Jacobson EE, Shaw J, Kaptchuk TJ (2012). Participants' experiences of being debriefed to placebo allocation in a clinical trial. Qual Health Res.

[15] Di Blasi Z, Crawford F, Bradley C, Kleijnen J (2005). Reactions to treatment debriefing among the participants of a placebo controlled trial. BMC Health Serv Res.

[16] Chen G, Johnson MH (2009). Patients' attitudes to the use of placebos: results from a New Zealand survey. N Z Med J.

[17] Berthelot JM, Maugars Y, Abgrall M, Prost A (2001). Interindividual variations in beliefs about the placebo effect: a study in 300 rheumatology inpatients and 100 nurses. Joint Bone Spine.

[18] Hughes J, Greville-Harris M, Graham CA, Lewith G, White P, Bishop FL (2017). What trial participants need to be told about placebo effects to give informed consent: a survey to establish existing knowledge among patients with back pain. J Med Ethics.

[19] Keränen T, Halkoaho A, Itkonen E, Pietilä A (2015). Placebo-controlled clinical trials: how trial documents justify the use of randomisation and placebo. BMC Med Ethics.

[20] Hernández A, Baños J, Llop C, Farré M (2014). The definition of placebo in the informed consent forms of clinical trials. PLoS One.

[21] Cheon S, Park H, Chae Y, Lee H (2018). Does different information disclosure on placebo control affect blinding and trial outcomes? A case study of participant information leaflets of randomized placebo-controlled trials of acupuncture. BMC Med Res Methodol.

[22] Kisaalita NR, Hurley RW, Staud R, Robinson ME (2016). Placebo use in pain management: a mechanism-based educational intervention enhances placebo treatment acceptability. J Pain.

[23] Bishop FL, McGinn L, Graham C, Biggs H, Denegri S, Lewith GT (2016). Describing placebos in patient information leaflets: effects on patients' beliefs. Eur J Pers Cent Healthc.

[24] Santana S, Lausen B, Bujnowska-Fedak M, Chronaki CE, Prokosch H, Wynn R (2011). Informed citizen and empowered citizen in health: results from an European survey. BMC Fam Pract.

[25] Atkinson NL, Saperstein SL, Pleis J (2009). Using the internet for health-related activities: findings from a national probability sample. J Med Internet Res.

[26] Cline RJ, Haynes KM (2001). Consumer health information seeking on the Internet: the state of the art. Health Educ Res.

[27] Cook DA, Dupras DM (2004). A practical guide to developing effective web-based learning. J Gen Intern Med.

[28] Greville-Harris M, Bostock J, Din A, Graham CA, Lewith G, Liossi C, O'Riordan T, White P, Yardley L, Bishop FL (2016). Informing patients about placebo effects: using evidence, theory, and qualitative methods to develop a new website. JMIR Res Protoc.

[29] Mistiaen P, van Osch M, van Vliet L, Howick J, Bishop FL, DiBlasi Z, Bensing J, van Dulmen S (2015). The effect of patient-practitioner communication on pain: a systematic review. Eur J Pain.

[30] Zhang W, Robertson J, Jones AC, Dieppe PA, Doherty M (2008). The placebo effect and its determinants in osteoarthritis: meta-analysis of randomised controlled trials. Ann Rheum Dis.

[31] Benedetti F (2008). Mechanisms of placebo and placebo-related effects across diseases and treatments. Annu Rev Pharmacol Toxicol.

[32] Hartvigsen J, Hancock MJ, Kongsted A, Louw Q, Ferreira ML, Genevay S, Hoy D, Karppinen J, Pransky G, Sieper J, Smeets RJ, Underwood M, Lancet Low Back Pain Series Working Group (2018). What low back pain is and why we need to pay attention. Lancet.

[33] GBD 2015 Disease and Injury Incidence and Prevalence Collaborators (2016). Global, regional, and national incidence, prevalence, and years lived with disability for 310 diseases and injuries, 1990-2015: a systematic analysis for the Global Burden of Disease Study 2015. Lancet.

[34] Foster NE, Anema JR, Cherkin D, Chou R, Cohen SP, Gross DP, Ferreira PH, Fritz JM, Koes BW, Peul W, Turner JA, Maher CG, Lancet Low Back Pain Series Working Group (2018). Prevention and treatment of low back pain: evidence, challenges, and promising directions. Lancet.

[35] Carvalho C, Caetano JM, Cunha L, Rebouta P, Kaptchuk TJ, Kirsch I (2016). Open-label placebo treatment in chronic low back pain: a randomized controlled trial. Pain.

[36] Brett J, Staniszewska S, Mockford C, Herron-Marx S, Hughes J, Tysall C, Suleman R (2014). Mapping the impact of patient and public involvement on health and social care research: a systematic review. Health Expect.

[37] Faul F, Erdfelder E, Lang AG, Buchner A (2007). G*Power 3: a flexible statistical power analysis program for the social, behavioral, and biomedical sciences. Behav Res Methods.

[38] Williams S, Yardley L, Wills GB (2013). A qualitative case study of LifeGuide: users' experiences of software for developing Internet-based behaviour change interventions. Health Informatics J.

[39] Coulter A, Ellins J (2006). The Health Foundation.

[40] Michie S, Abraham C (2004). Interventions to change health behaviors: evidence-based or evidence-inspired?. Psychol Health.

[41] Michie S, Johnston M (2012). Theories and techniques of behaviour change: developing a cumulative science of behaviour change. Health Psychol Rev.

[42] Yardley L, Morrison L, Bradbury K, Muller I (2015). The person-based approach to intervention development: application to digital health-related behavior change interventions. J Med Internet Res.

[43] Price DD, Finniss DG, Benedetti F (2008). A comprehensive review of the placebo effect: recent advances and current thought. Annu Rev Psychol.

[44] Ajzen I (1991). The theory of planned behavior. Organ Behav Hum Decis Process.

[45] Deyo RA, Dworkin SF, Amtmann D, Andersson G, Borenstein D, Carragee E, Carrino J, Chou R, Cook K, DeLitto A, Goertz C, Khalsa P, Loeser J, Mackey S, Panagis J, Rainville J, Tosteson T, Turk D, Von KM, Weiner DK (2014). Report of the NIH Task Force on research standards for chronic low back pain. J Pain.

[46] Marteau TM, Dormandy E, Michie S (2001). A measure of informed choice. Health Expect.

[47] Dormandy E, Hooper R, Michie S, Marteau TM (2002). Informed choice to undergo prenatal screening: a comparison of two hospitals conducting testing either as part of a routine visit or requiring a separate visit. J Med Screen.

[48] Michie S, Dormandy E, Marteau TM (2002). The multi-dimensional measure of informed choice: a validation study. Patient Educ Couns.

[49] Francis J, Eccles M, Johnston M, Walker A, Grimshaw J, Foy R, Kaner E, Smith L, Bonetti D (2004). City Research Online.

[50] Dima A, Lewith GT, Little P, Moss-Morris R, Foster NE, Hankins M, Surtees G, Bishop FL (2015). Patients' treatment beliefs in low back pain: development and validation of a questionnaire in primary care. Pain.

[51] Tabachnick BG, Fidell LS (2001). Using Multivariate Statistics.

[52] Van Breukelen GJ (2006). ANCOVA versus change from baseline: more power in randomized studies, more bias in nonrandomized studies [corrected]. J Clin Epidemiol.

[53] Patel SM, Stason WB, Legedza A, Ock SM, Kaptchuk TJ, Conboy L, Canenguez K, Park JK, Kelly E, Jacobson E, Kerr CE, Lembo AJ (2005). The placebo effect in irritable bowel syndrome trials: a meta-analysis. Neurogastroenterol Motil.

[54] Kirsch I, Deacon BJ, Huedo-Medina TB, Scoboria A, Moore TJ, Johnson BT (2008). Initial severity and antidepressant benefits: a meta-analysis of data submitted to the Food and Drug Administration. PLoS Med.

[55] Kaptchuk TJ, Friedlander E, Kelley JM, Sanchez MN, Kokkotou E, Singer JP, Kowalczykowski M, Miller FG, Kirsch I, Lembo AJ (2010). Placebos without deception: a randomized controlled trial in irritable bowel syndrome. PLoS One.

[56] Jonas WB, Crawford C, Colloca L, Kaptchuk TJ, Moseley B, Miller FG, Kriston L, Linde K, Meissner K (2015). To what extent are surgery and invasive procedures effective beyond a placebo response? A systematic review with meta-analysis of randomised, sham controlled trials. BMJ Open.

[57] Kwakye IN, Garner M, Baldwin DS, Bamford S, Pinkney V, Bishop FL (2016). Altruism, personal benefit, and anxieties: a phenomenological study of healthy volunteers' experiences in a placebo-controlled trial of duloxetine. Hum Psychopharmacol.

[58] Papadopoulos D, Mitsikostas DD (2012). A meta-analytic approach to estimating nocebo effects in neuropathic pain trials. J Neurol.

[59] Geraghty AW, Kirby S, Essery R, Little P, Bronstein A, Turner D, Stuart B, Andersson G, Carlbring P, Yardley L (2014). Internet-based vestibular rehabilitation for adults aged 50 years and over: a protocol for a randomised controlled trial. BMJ Open.

[60] Little P, Stuart B, Francis N, Douglas E, Tonkin-Crine S, Anthierens S, Cals JW, Melbye H, Santer M, Moore M, Coenen S, Butler C, Hood K, Kelly M, Godycki-Cwirko M, Mierzecki A, Torres A, Llor C, Davies M, Mullee M, O'Reilly G, van der Velden A, Geraghty AW, Goossens H, Verheij T, Yardley L (2013). Effects of internet-based training on antibiotic prescribing rates for acute respiratory-tract infections: a multinational, cluster, randomised, factorial, controlled trial. Lancet.

[61] Office for National Statistics (2015). Office for National Statistics.

[62] Sheeran P, Webb TL (2016). The intention-behavior gap. Soc Personal Psychol Compass.

