# Supplementary material for: Informing Adults With Back Pain About Placebo Effects: Randomized Controlled Evaluation of a New Website With Potential to Improve Informed Consent in Clinical Research
Source: J Med Internet Res. 2019 Jan 17;21(1):e9955. doi: 10.2196/jmir.9955 (PMC6354200; doi:10.2196/jmir.9955)
Supplement: Multimedia Appendix 1 [file jmir_v21i1e9955_app1.pdf]

Multimedia Appendix 1

Description of the Factorial Trial Design

In the larger trial, the two factors were website topic (placebo vs acupuncture) and website quality (‘person-based’ vs ‘control’). Participants were randomised using simple randomisation implemented by the website to one of four groups, representing every combination of the two factors. Every participant therefore viewed two websites: one about placebo (‘person-based’ or ‘control’), and one about acupuncture (‘person-based’ or ‘control’). The order of the websites was counterbalanced, so some participants saw a placebo website first, others saw an acupuncture website first (see Table). There were no interaction effects between placebo and acupuncture websites and no effects of acupuncture website quality on placebo-related outcomes (e.g. main effect of acupuncture website on placebo knowledge: $F(1, 327)=2.535$, $p=.112$; interaction effect of acupuncture and placebo websites on placebo knowledge: $F(1,327)=0.120$, $p=.729$). Therefore, the results of the person-based vs control comparison for the acupuncture website are being reported separately.

Table: Factorial Design

	Placebo website quality	Acupuncture website quality
Group 1	Person-based	Person-based
Group 2	Standard	Person-based
Group 3	Person-based	Standard
Group 4	Standard	Standard

Note. This paper reports the comparison between person-based and standard placebo websites: groups 1 and 3 were collapsed and groups 2 and 4 were collapsed.
