# Supplementary material for: Informing Adults With Back Pain About Placebo Effects: Randomized Controlled Evaluation of a New Website With Potential to Improve Informed Consent in Clinical Research
Source: J Med Internet Res. 2019 Jan 17;21(1):e9955. doi: 10.2196/jmir.9955 (PMC6354200; doi:10.2196/jmir.9955)
Supplement: Multimedia Appendix 2 [file jmir_v21i1e9955_app2.pdf]

Supplemental Digital Content

Screenshots from person-based website about placebo effects

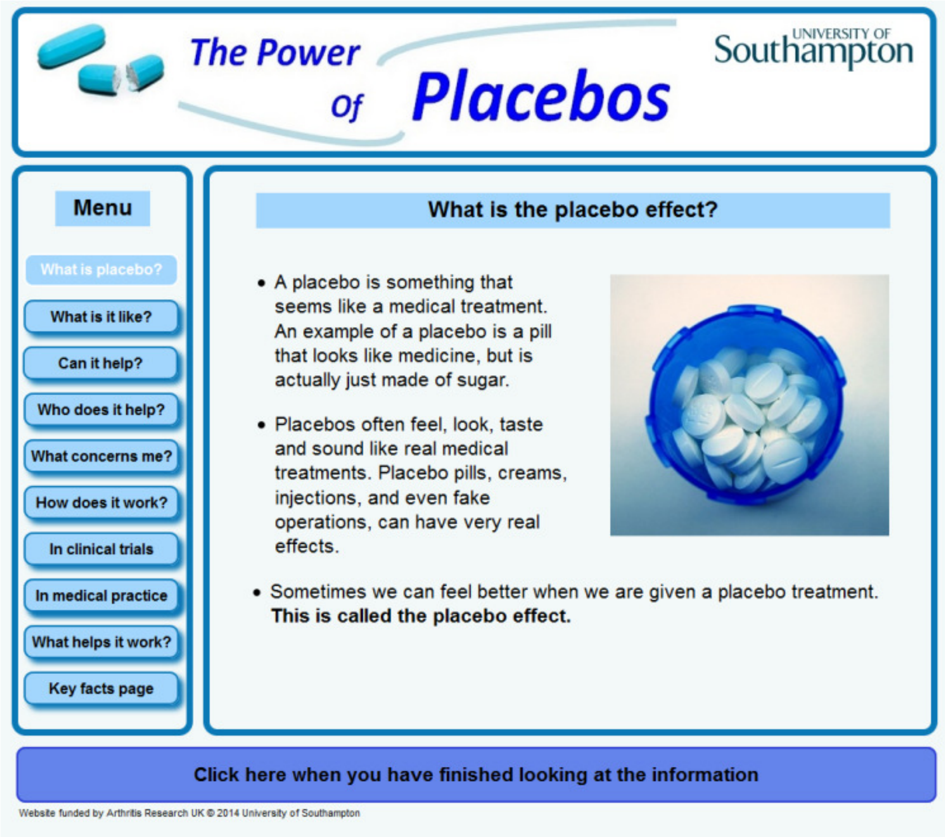

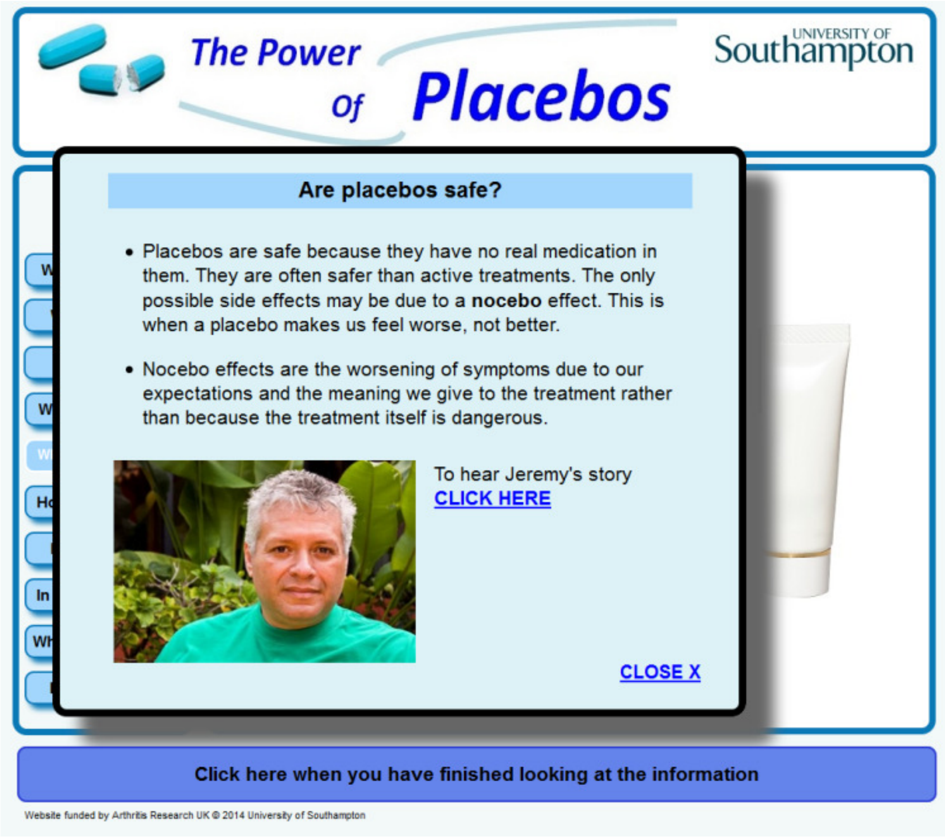

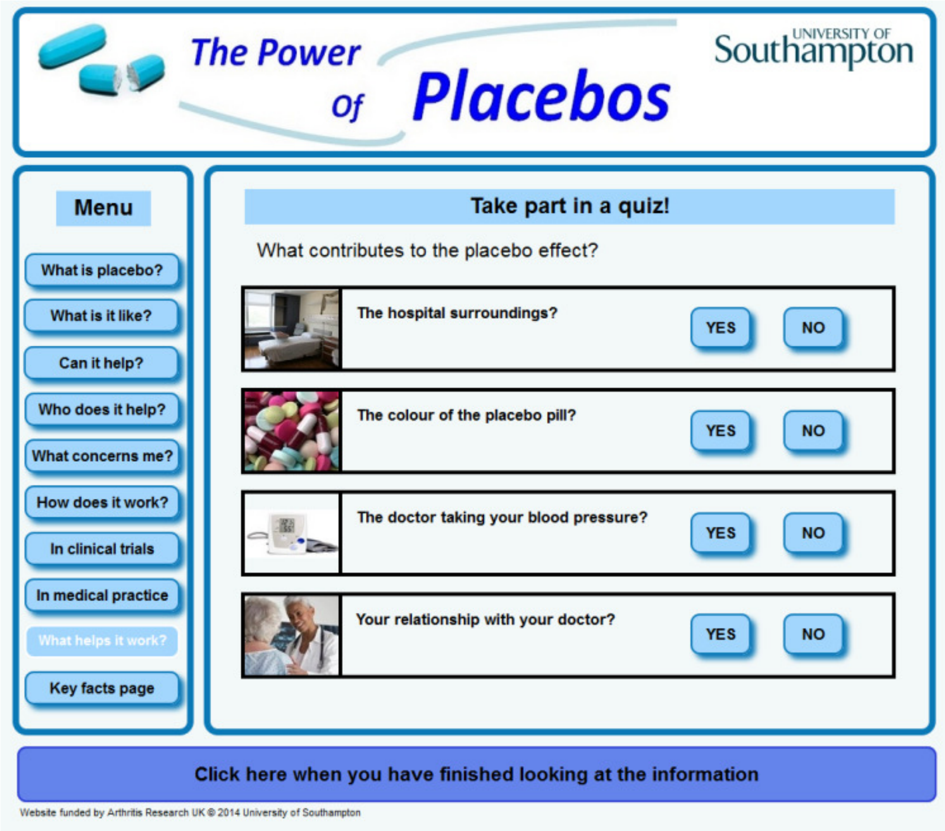
The Power Of Placebos
UNIVERSITY OF Southampton
Menu
What is placebo?
What is it like?
Can it help?
Who does it help?
What concerns me?
How does it work?
In clinical trials
In medical practice
What helps it work?
Key facts page
Take part in a quiz!
What contributes to the placebo effect?
The hospital surroundings?
YES
NO
The colour of the placebo pill?
YES
NO
The doctor taking your blood pressure?
YES
NO
Your relationship with your doctor?
YES
NO
Click here when you have finished looking at the information
Website funded by Arthritis Research UK © 2014 University of Southampton

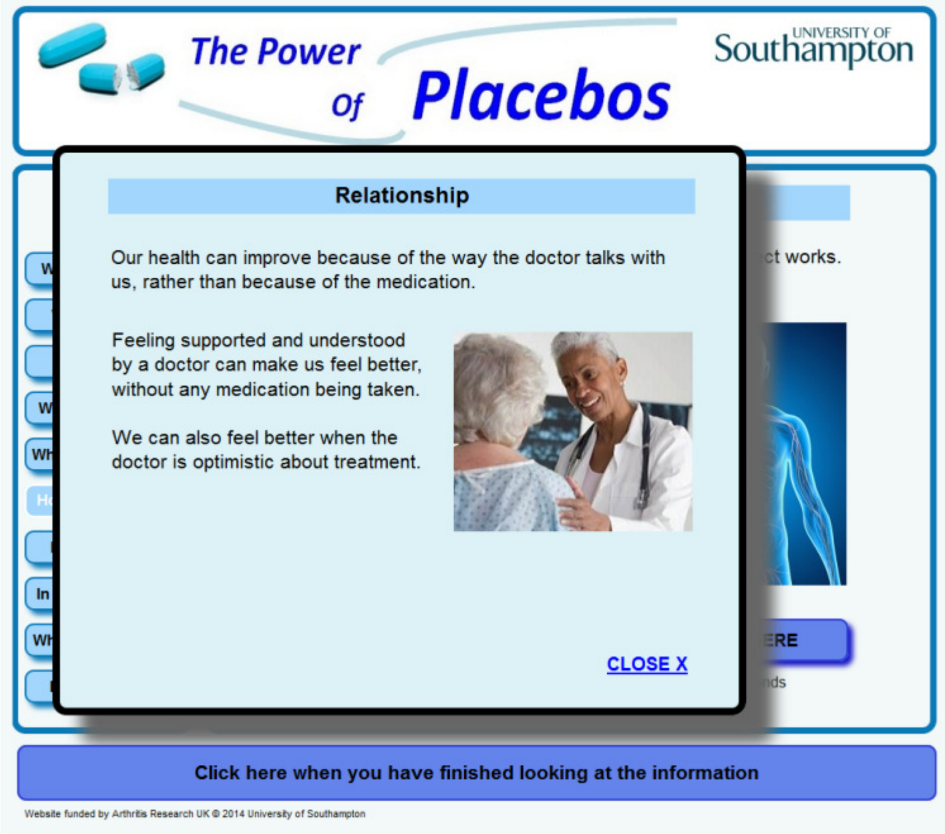
The Power Of Placebos
UNIVERSITY OF Southampton
Relationship
Our health can improve because of the way the doctor talks with us, rather than because of the medication.
Feeling supported and understood by a doctor can make us feel better, without any medication being taken.
We can also feel better when the doctor is optimistic about treatment.
CLOSE X
Click here when you have finished looking at the information
Website funded by Arthritis Research UK © 2014 University of Southampton
