# Supplementary material for: Informing Adults With Back Pain About Placebo Effects: Randomized Controlled Evaluation of a New Website With Potential to Improve Informed Consent in Clinical Research
Source: J Med Internet Res. 2019 Jan 17;21(1):e9955. doi: 10.2196/jmir.9955 (PMC6354200; doi:10.2196/jmir.9955)
Supplement: Multimedia Appendix 4 [file jmir_v21i1e9955_app4.pdf]

# Placebos in medical trials

**Menu**

- What is a placebo?
- What are placebo-controlled trials?
- Would I know if I was taking a placebo?
- What happens when a trial ends?

UNIVERSITY OF Southampton

## What is a placebo?

A placebo treatment contains no medication but looks, tastes, and/or feels like a medical treatment.

Placebo treatments are often thought of as pills or tablets. Other types of placebos called “sham treatments” are used to test treatments like physiotherapy or surgery.

We cannot tell you what is in a placebo, because the ingredients in a placebo are different in different trials. For example, some trials use indigestible tablets made of starch and some use injections of saline (which is salt water).

Placebos are inert. Placebos contain no active ingredients. Some people might feel a bit better just from taking part in a placebo-controlled clinical trial.

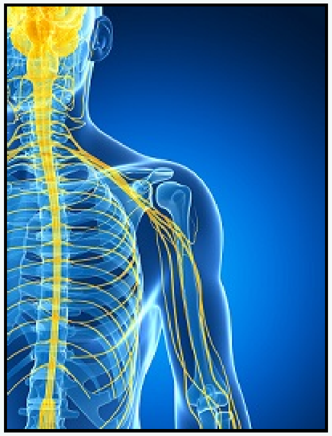

**Key fact**

**A placebo treatment is a dummy treatment.**

**Click here when you have finished looking at the information**

Website funded by Arthritis Research UK © 2014 University of Southampton

# Placebos in medical trials

**Menu**

What is a placebo?

What are placebo-controlled trials?

Would I know if I was taking a placebo?

What happens when a trial ends?

UNIVERSITY OF Southampton

## What are placebo-controlled trials?

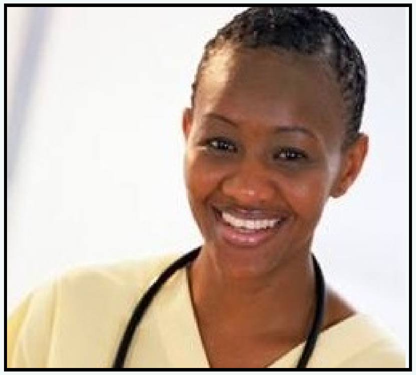

Sometimes doctors don't know whether a medicine works. To find out, researchers compare the medicine to a placebo. They carry out a placebo-controlled clinical trial.

Placebo-controlled clinical trials aim to find out:

- If the medicine works better than a placebo.
- How well the medicine works and how long the effects last.
- How common or serious any side effects are.

CLICK HERE to find out about group randomisation.

**Click here when you have finished looking at the information**

Website funded by Arthritis Research UK © 2014 University of Southampton

# Placebos in medical trials



Southampton UNIVERSITY OF

## Randomisation

In a placebo-controlled clinical trial, researchers put people into 2 groups. One group takes the medicine. The other group takes placebos. To try to make the groups the same to start with, each patient is put into a group by chance. This is called randomisation and is usually done by a computer.

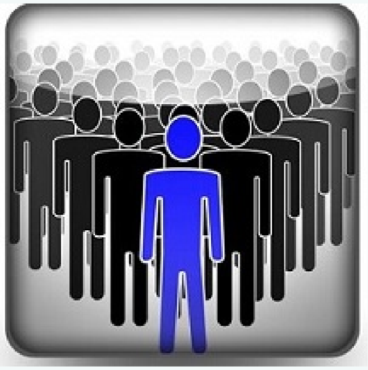

Randomisation means that each group has a similar mix of people of different ages, gender, and health.

At the end of the trial the researchers compare the health of the two groups. If the medicine group does better than the placebo group, it is likely to be because of the medicine. This is because the two groups are very similar in every other way.



Website funded by Arthritis Research UK © 2014 University of Southampton

# Placebos in medical trials

Menu

What is a placebo?

What are placebo-controlled trials?

Would I know if I was taking a placebo?

What happens when a trial ends?

UNIVERSITY OF Southampton

## Would I know if I was taking a placebo?

This depends on what type of trial you take part in. Most placebo-controlled trials are either "single-blind" or "double-blind" trials.

In a "single-blind trial" you will not know whether you are taking the medicine or the placebo. This is because if patients know which treatment they are getting this might influence how they feel or how they report their symptoms.

In a "double-blind trial" neither you nor your doctor will know whether you are taking the medicine or the placebo. This helps to prevent the doctors' expectations from influencing the results. Your doctor can find out which treatment you are taking if necessary.

In all trials patients are very carefully monitored and treatment is stopped if necessary.

**Did you know?**

To prevent people from guessing which treatment they are getting, placebos are designed to look exactly like the medicine in the trial.

**Click here when you have finished looking at the information**

Website funded by Arthritis Research UK © 2014 University of Southampton

# Placebos in medical trials

Menu

What is a placebo?

What are placebo-controlled trials?

Would I know if I was taking a placebo?

What happens if a trial ends?

University of Southampton

## What happens when a trial ends?

In medical trials, patients are usually told whether or not the new medicine was effective. Sometimes patients find out whether they have been taking the new medicine or the placebo. Sometimes patients are able to take the new medicine.

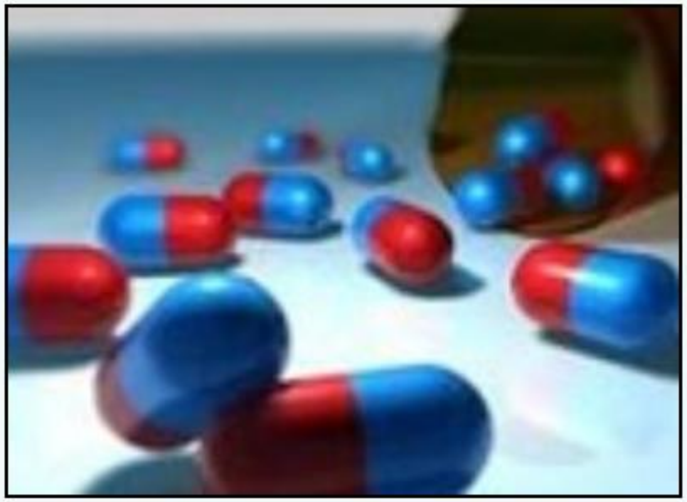

**Key fact**

**Placebos are fake treatments.**

**Click here when you have finished looking at the information**

Website funded by Arthritis Research UK © 2014 University of Southampton
