# Supplementary material for: Informing Adults With Back Pain About Placebo Effects: Randomized Controlled Evaluation of a New Website With Potential to Improve Informed Consent in Clinical Research
Source: J Med Internet Res. 2019 Jan 17;21(1):e9955. doi: 10.2196/jmir.9955 (PMC6354200; doi:10.2196/jmir.9955)
Supplement: Multimedia Appendix 5 [file jmir_v21i1e9955_app5.pdf]

Information sheet about our study- providing information about acupuncture and placebos online

What is the research about?

Lots of people use the internet to find out more information, and we are interested in finding out whether our different online information pages about placebos and acupuncture are useful and informative. We are also interested in finding out how we can develop our information pages in the future, and how they can be improved. The aim of this study therefore, is to find out how useful the different information pages that we have developed are. We will be asking you to look at some information about acupuncture and some information about placebos as part of this study. We will also ask you to fill in some questions about yourself, your back pain and your thoughts about placebos and acupuncture.

What will happen if I decide to take part?

If you decide to take part in this study, you will be asked to login to our study online. To do this, you will need to enter your email address and create a password. You will then be able to log in and out of the study as many times as you would like, as long as you remember your email and password. This means that you can complete the study in stages if that is more convenient for you.

You will be asked to answer some questions about acupuncture and placebos. We will then show you some information about acupuncture and placebos online. Because the study shows images, descriptions and footage of needles, **please do not sign up to take part if you have needle phobia.**

You will either see shorter information pages about acupuncture and placebos, or longer information pages, depending on which group you are randomly assigned to. After you have looked at these pages we will ask you to complete some more questionnaires. These questionnaires will ask you questions about your back pain as well as some questions about yourself (such as your age and ethnicity). You will then be asked some more questions about acupuncture and placebos. The whole study should take between 60 and 120 minutes to complete, depending on which information pages we ask you to look at. We will be remotely monitoring which pages you view, and for how long, whilst you are doing this study.

Why have I been invited?

You have been invited to take part because you have back pain or have had an episode of back pain in the last three years.

Do I have to take part?

Taking part in this study is completely voluntary. Whether you decide to take part or not will not affect your future medical care in any way. If you do decide to take part, you can also withdraw from the study at any time by closing the web browser.

Are there any possible risks involved?

Some people find some of the questionnaires for this study a bit personal or sensitive, particularly questions about your back pain. If you do not wish to answer any of the questions, please feel free to leave them blank and move on. If you do find the study upsetting, please feel free to withdraw from the study at any time by closing your web browser. If the questions about your back pain do bring up any health concerns for you, please contact your GP or NHS Choices: http://www.nhs.uk/Pages/HomePage.aspx. If you have any more immediate health concerns, we advise you to contact NHS 111.

If you are scared of needles you may be alarmed by images, text and film content related to acupuncture treatment in this study. **If you do have needle phobia, please do not take part in this study.**

Our information pages will not advise you to change any medication that you are currently taking. If you do wish to change your medication or treatment for your back pain, we advise that you discuss this with your GP first.

This study involves looking at information online and filling out questionnaires at your computer. If you sit at a computer for a long period of time, this may aggravate your back pain. We encourage you to take regular breaks throughout this study. You may like to complete the study in several sections. If so, you can log out of the website and log in again at a later date by entering your email and password. When you log in again, you will be taken back to the section that you last viewed so that you can carry on where you left off. Please do not complete this study on your mobile phone.

Some of the quiz questions about treatments at the end of this study are quite difficult. This is not a test of your IQ and it is very normal to be unsure of some of the answers to these questions.

What are the benefits of taking part?

Participants who complete this study will be emailed a £10 Amazon voucher for completing this study. The research team will be able to record which pages of the study you have viewed and will email you a voucher shortly after you have completed the study.

Some people may find the information in this study interesting and informative. If you would like to learn more about acupuncture and placebos, we will also provide links to more information at debriefing.

How do I take part?

This study will be running until December 2014 so you can decide at any point up until then whether you would like to take part in the study. To take part, please follow this web link: (ENTER LINK,) or

google (ENTER SEARCH TERM), or contact the researcher at email: M.L.Greville-Harris@soton.ac.uk. If you decide to take part, please complete this study on a computer, as this study is not designed to be completed using mobile phones.

What if there is a problem?

If there is a problem you can contact the researcher for this study by email at: M.L.Greville-Harris@soton.ac.uk. If you have any questions about your rights as a participant in this research, or if you feel that you have been placed at risk, you can contact the chair of the Ethics Committee, Psychology, University of Southampton, SO17 1BJ, UK. Phone: 023 8059 4663, Email: fshs-rso@soton.ac.uk. **If after contacting the University, you still feel that your complaint has not been dealt with satisfactorily, you can also make use of the NHS Complaints System (Patient Advice and Liaison Service, PALS, Southampton NHS treatment Centre) by contacting pals@scpct.nhs.uk, phone 023 8029 6929.**

Will my taking part in the study be kept confidential?

We will ask you to enter your email address for this study. Your email address will only be used to contact you if you forget your password, to send you a reminder about our study if you complete it in sections, or to contact you if you have difficulties logging on to the study website. Your email address will be separated from the data at the earliest opportunity. All data for this study will be stored on a University of Southampton password protected computer. Data will be secured securely at the University of Southampton for 10 years before being destroyed. None of the results from this study will have any identifiable information in it, and all of your responses will be kept confidential.

What will happen to the results of the research study?

The results of this research study will be passed on to Arthritis Research UK and the findings from the study will be used for publication or educational purposes. Any information that we use will not identify anyone who took part in the study. A summary of the results will be available from March 2015. Please email the researcher M.L.Greville-Harris@soton.ac.uk if you would like to receive a summary of the results for this study.

Who is organizing and funding the research?

This research is being carried out at the University of Southampton. The project is funded by Arthritis Research UK.

For further information contact:

Dr Maddy Greville-Harris

M.L.Greville-Harris@soton.ac.uk

Telephone: 0238 0595 748

Room 4067, Building 44

University of Southampton

Highfield Campus

SO17 1BJ

Statement of Consent

Please tick the boxes next to each statement if you agree:

I certify that I am 18 years or older ☐

I certify that I do not suffer from a needle phobia ☐

I understand that data collected as part of this research will be kept confidential and that published results will maintain that confidentiality. I understand that I may withdraw from the study at any time without penalty. I also understand that data collected during the study, may be looked at by individuals from the University of Southampton, from regulatory authorities or from the NHS trust, where it is relevant to my taking part in this research. I give permission for these individuals to have access to my records. ☐

I understand that if I have any questions about my rights as a participant in this research, or if I feel that I have been placed at risk, I may contact the chair of the Ethics Committee, Psychology, University of Southampton, SO17 1BJ, UK. Phone: 023 8059 4663, email: fshs-rso@soton.ac.uk. I understand that if I feel that my complaint has not been dealt with, I can also make use of the NHS Complaints System (Patient Advice and Liaison Service by contacting pals@scpct.nhs.uk, phone 023 8029 6929. ☐

I have read the information sheet about this research project. I give consent to participate in the above described research. In consenting, I understand that my legal rights are not affected. ☐
